# Alcohol Use and Cancers of the Gastrointestinal Tract. Epidemiology and Preventive Implications

**DOI:** 10.3389/fonc.2020.00403

**Published:** 2020-03-25

**Authors:** Jürgen Rehm, Kevin Shield

**Affiliations:** ^1^Centre for Addiction and Mental Health (CAMH), Institute for Mental Health Policy Research, Toronto, ON, Canada; ^2^Dalla Lana School of Public Health, University of Toronto, Toronto, ON, Canada; ^3^Centre for Addiction and Mental Health, Campbell Family Mental Health Research Institute, Toronto, ON, Canada; ^4^Department of Psychiatry, University of Toronto, Toronto, ON, Canada; ^5^Institute of Clinical Psychology and Psychotherapy, Faculty of Psychology, School of Science, Technische Universität Dresden, Dresden, Germany; ^6^Department of International Health Projects, Institute for Leadership and Health Management, I.M. Sechenov First Moscow State Medical University, Moscow, Russia

**Keywords:** alcohol, gastrointestinal tract, liver cancer, colorectal cancer, esophagus cancer, mortality, burden of disease, prevention

## Abstract

**Introduction:** Alcohol is a carcinogen for human cancer. This contribution summarizes the relationships between alcohol use and gastrointestinal cancers, and implications for prevention.

**Methods:** Comparative risk assessment and narrative literature review.

**Results:** The following gastrointestinal cancer sites were found to be causally impacted by alcohol use: lip and oral cavity, pharynx other than nasopharynx, esophagus, colon and rectum, and liver. Globally, 368,000 deaths (304,000 men and 64,000 women) and more than 10 million disability-adjusted life years (DALYs) lost (10.1 million; 8.4 million men and 1.6 million women) in 2016 were attributable to alcohol use, making up about 10% of all deaths and DALYs lost due to these cancers, respectively. There are effective and cost-effective alcohol control policies available to reduce this burden, namely the best buys of increasing taxation, reducing availability, and banning advertisement. In addition, public knowledge about the alcohol-cancer link should be increased.

**Discussion:** There are a number of assumptions underlying these estimates, but overall all of them seem to be conservative.

## Introduction

Alcohol use is one of the major risk factors for cancer (1, 2), with about 5% of all cancers being caused by alcohol (3). Gastrointestinal cancers, which contributed about one third to all cancer burden of disease and more than 40% to all cancer deaths in 2017 (4), are no exception. On the contrary, alcohol-attributable gastrointestinal cancer types made up the majority of all alcohol-attributable cancers [more than 80% of both mortality and burden of disease (3)].

For most of the cancers in the gastrointestinal tract, alcohol use has been established as a causal factor [for criteria see (5, 6)], and the categorizations converge between the International Agency for Research on Cancer (7, 8) and the Continuous Update Project of the World Cancer Research Fund (9) (see Table 1 for details).

**Table 1:** Alcohol-attributable mortality and burden of disease for cancers of the gastrointestinal tract in 2016.

**Cancer type**	**Deaths (in thousands)**	**DALYs (in millions)**	**PAF in %**
Lip and oral cavity cancer	52.2	1.7	31.3
Pharynx other than nasopharynx	38.6	1.2	34.9
Esophagus	82.6	2.2	19.3
Colon and rectum	92.6	2.2	11.7
Liver	101.4	2.8	12.2
All cancers of the gastrointestinal tract	367.7	10.1	9.9

*PAF, population-attributable fraction*.

It is the aim of this contribution to give epidemiological indicators for alcohol-attributable cancer burden for organs of the gastrointestinal tract globally, and to look into the best prevention efforts. As a basis, we used a recent publication on alcohol use and disease burden (3), from which we aggregated the alcohol-attributable death and burden of disease [in disability-adjusted life-years—DALYs—lost (10)], and the recommendations of the World Health Organization (WHO) (11) to prevent premature mortality from non-communicable disease, namely the so-called “best buys,” which constitute a particular set of alcohol control mechanism, which are not only highly cost-effective (12), but also very affordable and easy to implement (13).

## Materials and Methods

Based on available global data (14), the following cancer categories were considered to be in part caused by alcohol use: lip and oral cavity (C00–C08), other pharynx than nasopharynx (C12–C14), esophagus (C15), colon and rectum (C18–C21), and liver (C22). Thus, our estimates exclude cancers of small intestine (C17) and cancer of the intestinal tract, part unspecified (C26), which has been determined to be potentially alcohol-attributable (7, 8).

The mortality and disease burden of alcohol-attributable gastrointestinal cancer for 2016 was calculated based on the recent publication of Shield et al. (3), who based their estimates on the exposure estimates of Manthey et al. (15) combined with cancer-site specific risk relations of Turati et al. (16) for liver cancer, and Bagnardi et al. (17) for all other cancer categories. A 10-year lag between exposure and outcomes was assumed (18).

Shield and colleagues performed a comparative risk assessment for alcohol (19) based on the tradition of the Global Burden of Disease Studies [last assessment published: (20) and the WHO Global Status Reports on Alcohol and Health [last: (21)]. The usual formula for attributable risk was used (22), with exposure being continuous, with a specific term for former drinkers added (23, 24) to adjust for the “sick quitter” phenomenon (25)]. For a general discussion of the assumptions of comparative risk assessments, see the overview by Murray et al. (26), and for further detail on the assumptions for comparative risk assessments for alcohol, [see Rehm et al. (27)]. All dose-response curves show a monotonous exponential course (3), with cancers of the upper digestive tract showing steeper slopes. Further details on the risk relation formulas can be found in Shield et al. (3).

Prevention suggestions were based on the WHO's recommendations for prevention and control of non-communicable disease burden (11), with special emphasis on the cost-effectiveness of models.

## Results

### Epidemiology

Globally, 368,000 deaths (304,000 men and 64,000 women) and more than 10 million DALYs lost (10.1 million; 8.4 million men and 1.6 million women) in 2016 were attributable to alcohol use, making up about 10% of all deaths and DALYs lost due to these cancers, respectively. These deaths and years of life lost could have been avoided if no alcohol had been consumed. Table 2 gives an overview of alcohol-attributable mortality and burden of disease by subcategory of gastrointestinal cancer.

**Table 2:** Alcohol-attributable mortality and burden of disease for cancers of the gastrointestinal tract in 2016.

**Cancer type**	**Deaths thousands (95% CI)**	**DALYs lost millions (95% CI)**	**PAF in % (95% CI)**
Lip and oral cavity cancer	52.2 (35.2, 53.1)	1.7 (1.1, 1.7)^*^	31.3 (21.1, 31.8)
Pharynx other than nasopharynx	38.6 (26.6, 40.1)	1.2 (0.8, 1.2)^*^	34.9 (24.1, 36.3)
Esophagus	82.6 (55.3, 85.7)	2.2 (1.5, 2.2)^*^	19.3 (12.9, 20.0)
Colon and rectum	92.6 (73.7, 109.1)	2.2 (1.8, 2.6)	11.7 (9.3, 13.7)
Liver	101.4 (54.1, 140.4)	2.8 (1.5, 3.9)	12.2 (6.5, 16.9)
All cancers of the gastrointestinal tract	367.7 (278.6, 394.1)	10.1 (7.5, 10.7)	9.9 (7.5, 10.7)

*CI, Confidence interval; DALYs, disability-adjusted life years; PAF, population-attributable fraction of burden of disease in DALYs lost*.

* *Due to the dose-response function, the 95% confidence intervals are asymmetric*.

**Figure 1:**
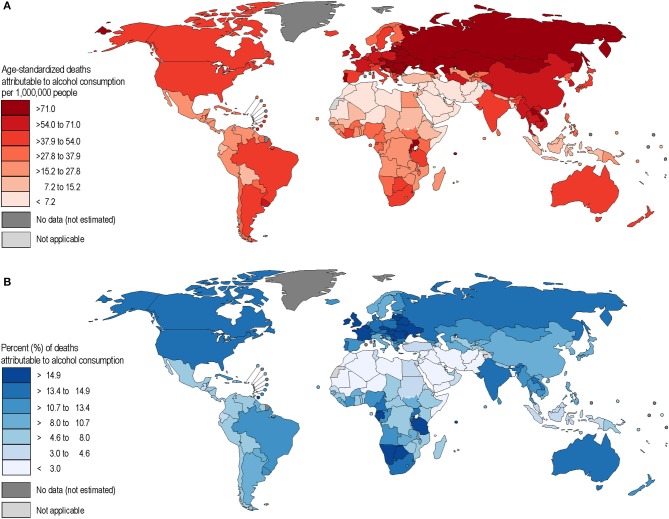
**(A,B)** Age-standardized mortality rates and population-attributable fractions of alcohol-attributable gastrointestinal cancers in 2016.

**Figure 2:**
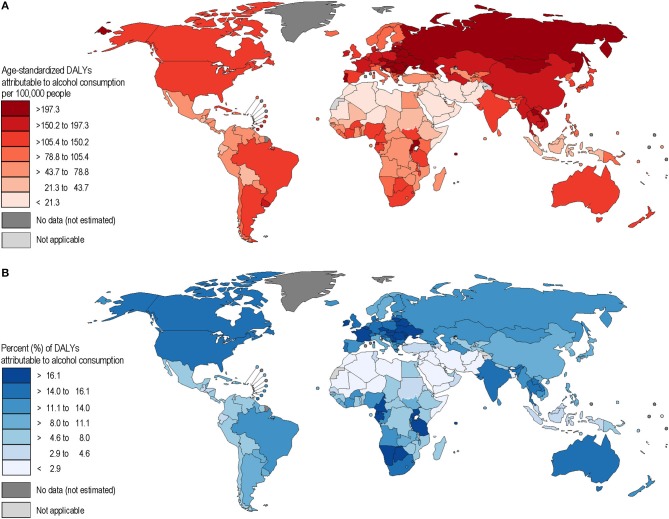
**(A,B)** Age-standardized disability-adjusted life years (DALY) rates and population-attributable fractions of alcohol-attributable gastrointestinal cancers in 2016.

Alcohol-attributable liver cancer had the largest share of mortality and burden of disease out of all the alcohol-attributable cancer categories, followed by colorectal cancers. Burden showed a high variation in different parts of the world, mainly based on the distribution of alcohol use (Figures 1A,B, 2A,B). It should be noted that all figures show point estimates only, and some of these estimates have wide confidence intervals (see also Table 2).

The highest alcohol-attributable cancer mortality and burden rates are found in eastern Europe, and parts of Central Asia, whereas countries in the Muslim belt from North Africa to Pakistan were found to have the lowest rates of mortality and burden (see Figures 1A,B, 2A,B).

### Prevention

As shown above, the burden of alcohol-attributable gastrointestinal cancer is large, with more than 360,000 deaths and more than one million DALYs lost. What are the best prevention strategies, based on the risk relations between alcohol use and gastrointestinal cancer? First, unlike other alcohol-attributable disease outcomes with complex risk curves for the average level of drinking and interactions with the pattern of drinking—such as ischemic heart disease (28, 29)—the relationship between gastrointestinal cancer and alcohol is simple: the more alcohol consumed, the higher the risk (3). Prevention efforts should therefore mainly focus on a reduction in the level of drinking (30). There also do not seem to be specific effects for different beverage types (31). Thus, the best ways to reduce overall level of consumption would be those that are effective in the prevention of any type of cancer, and for those which work to prevent gastrointestinal cancer specifically (32). Any reduction in alcohol consumption will be associated reductions in cancer risks, albeit with lag times [e.g., (33, 34)].

Given this situation, and the fact that many alcohol-attributable cancers are found light to moderate drinkers (35, 36), the most effective and cost-effective prevention method available to us is the alcohol control policy outlined in the WHO “best buys” [e.g., (12, 13): an increase in the price of alcoholic beverages via an increase in alcohol taxation (37)], and restrictions on their availability, such as a restriction on sale hours, and a ban on marketing (38). In addition, there may be two interventions to prevent alcohol-attributable gastrointestinal cancer burden specifically: educating the public and container labeling. Both of these interventions build on the fact that knowledge that alcohol is a carcinogen has still not entered into the mainstream in most countries (39). Even those who indicated an awareness of the impact alcohol use has on cancer, did not know whether the risk was specific to alcohol; rather, they viewed it as being part of an overall belief that “everything causes cancer” (40). This indicates an urgent need to inform the general public regarding the alcohol-cancer link. Given the marked risk of usual alcohol intake, one common way of disseminating this information would be via the use of specific cancer-warning labels on alcoholic-beverage packaging (41). While most evidence available regarding the effectiveness of warning labels placed on packaging is not specific to alcohol, we see no reason why such an intervention would not work for alcohol as well. There have, in fact, been some reports of positive effects from the use of warning labels for alcohol containers as well [e.g., (42)].

## Discussion

We would first like to highlight the potential limitations of our study. As for the epidemiology, almost all single studies have found a relationship between alcohol use and the risk of gastrointestinal cancers. Biological evidence corroborates these findings. While knowledge about the biological pathways of cancer is certainly not complete, acetaldehyde—the first derivative of alcohol metabolism in humans and an ingredient in some alcoholic beverages (43)—seems to be the major pathway between alcohol use and esophagus and other gastrointestinal cancers (8): Acetaldehyde associated with consumption of alcoholic beverages has been classified as a Group 1 carcinogen for humans [i.e., sufficient evidence exists that it is carcinogenic; see also (44, 45)].

Part of the evidence for acetaldehyde serving as a biological pathway in the development of cancer came from studies in populations having genetic constellations (Aldehyde Dehydrogenase 2 gene) that slowed the breakdown of acetaldehyde, thereby creating a situation in which acetaldehyde was active for longer periods of time (46). People with this genetic constellation had a markedly increased risk of esophagus and other gastrointestinal cancers (7).

The increased risk for people with different genetic constellations in some Asian populations has not yet been incorporated into the comparative risk assessments like those noted above. Comparative risk assessments to date have been based on risk relations found mainly in Western high-income countries, which makes the above analysis conservative, and suggests that the gastrointestinal cancer risks posed by alcohol use may be markedly underestimated in Asian countries (47).

All of these findings assumed a lag time between alcohol use and cancer of 10 years (18). Clearly, this is an average across individuals and cancer subtypes, which may lead to biases that are not quantified in the current analyses.

The most contested is that the finding that there is no lower threshold for the impact of alcohol use on cancer (7). While there are empirical studies which seem to show protective effects for light drinking, most of these studies cannot exclude the “sick quitter” hypothesis—that abstainers are former drinkers who gave up alcohol due to health problems [(25); see also (48)]. Indeed, there are significantly elevated risks for former drinkers for all gastrointestinal cancers (3).

Finally, focusing only on the prevention of gastrointestinal cancer may lead to biased recommendations since alcohol use has been linked to over 200 diseases and injuries with different risk relations (6, 49). While it is true that some of these diseases may require additional alcohol control measures specifically directed at certain patterns of drinking [e.g., (6)], reducing the overall volume of drinking will yield at least some benefit for all of these disease and injury categories.

In conclusion, alcohol use is a major contributor to gastrointestinal cancers globally, with the exception of some Muslim-majority countries. There are effective and cost-effective alcohol control policies to reduce alcohol-attributable burden available: most importantly, the WHO's three best buys (increase in taxation, restriction on availability, and ban on advertisement), in addition to public education efforts, including the placement of warning labels on alcoholic beverage containers to increase public awareness that alcohol is a carcinogen.

## Data Availability Statement

The datasets generated for this study are available on request to the corresponding author.

## Author Contributions

JR and KS are equally responsible for all parts of the article.

### Conflict of Interest

The authors declare that the research was conducted in the absence of any commercial or financial relationships that could be construed as a potential conflict of interest.
